# Reduced Albumin Concentration Predicts Weight Gain and Higher *Ad Libitum* Energy Intake in Humans

**DOI:** 10.3389/fendo.2021.642568

**Published:** 2021-03-11

**Authors:** Alessio Basolo, Takafumi Ando, Douglas C. Chang, Tim Hollstein, Jonathan Krakoff, Paolo Piaggi, Susanne Votruba

**Affiliations:** ^1^Obesity and Diabetes Clinical Research Section, Phoenix Epidemiology and Clinical Research Branch, National Institute of Diabetes and Digestive and Kidney Diseases, National Institutes of Health, Phoenix, AZ, United States; ^2^Unit of Endocrinology, Department of Clinical and Experimental Medicine, University Hospital of Pisa, Pisa, Italy; ^3^Japan Society for the Promotion of Science, Tokyo, Japan; ^4^Department of Information Engineering, University of Pisa, Pisa, Italy

**Keywords:** albumin, energy intake, feeding behavior, food intake, weight change, energy expenditure

## Abstract

**Objective:**

Circulating albumin is negatively associated with adiposity but whether it is associated with increased energy intake, lower energy expenditure or weight gain has not been examined.

**Methods:**

In **study 1** (n=238; 146 men), we evaluated whether fasting albumin concentration was associated with 24-h energy expenditure and *ad libitum* energy intake. In **study 2** (n=325;167 men), we evaluated the association between plasma albumin and change in weight and body composition.

**Results:**

After adjustment for known determinants of energy intake lower plasma albumin concentration was associated with greater total daily energy intake (β= 89.8 kcal/day per 0.1 g/dl difference in plasma albumin, p=0.0047). No associations were observed between plasma albumin concentrations and 24-h energy expenditure or 24-h respiratory quotient (p>0.2). Over 6 years, volunteers gained on average 7.5 ± 11.7 kg (p<0.0001). Lower albumin concentrations were associated with greater weight [β=3.53 kg, p=0.039 (adjusted for age, sex, follow up time), CI 0.16 to 6.21 per 1 g/dl difference albumin concentration] and fat mass (β=2.3 kg, p=0.022), respectively, but not with changes in fat free mass (p=0.06).

**Conclusions:**

Lower albumin concentrations were associated with increased *ad libitum* food intake and weight gain, indicating albumin as a marker of energy intake regulation.

**Clinical Trial Registration:**

ClinicalTrials.gov, identifiers NCT00340132, NCT00342732.

## Introduction

The imbalance between energy intake and expenditure leads to the development of obesity, a condition associated with various metabolic diseases (1) and low-grade chronic inflammation (2). The adipose tissue, as an endocrine organ (3), produces a variety of inflammatory markers (adipocytokines) such as tumor necrosis factor (TNF-α), interleukin 1 (IL-1) and interleukin 6 (IL-6) (4, 5). Concentrations of plasma IL-6, TNF- α and other inflammatory markers increases in proportion with the increase in body mass index (BMI) in adolescents and elderly individuals (6, 7). Importantly, inflammation may play a role in the regulation of energy balance (8). In mice, IL-6 reduced food intake and hyperphagia (9) and IL-6 and TNF- α increased energy expenditure (10).

Albumin has recently been reported as a valid marker of the inflammatory process (11, 12) although, historically, it has also been described as marker of protein malnutrition (13). Albumin is the most abundant circulating protein in healthy subjects representing nearly 50% of total protein content in the human body (11, 14). Under physiological conditions albumin is almost exclusively produced by the liver (15) and its synthesis is inhibited by proinflammatory molecules such as IL-6 and TNF- α (15). Reduced albumin levels are associated with higher percentage of body fat and adipose tissue inflammation markers such as macrophages (16), indicating that reduced albumin concentrations might be linked with the inflammatory process and development of obesity. Albumin binds ghrelin and may impact the orexigenic effects of this hormone, additionally indicating a role for albumin in appetite regulation (17).

Given its inverse association with adiposity, its positive association with markers of subclinical inflammation that may influence appetite and its role in binding ghrelin, we investigated whether albumin concentration might be a determinant of factors that affect energy balance including 24-h energy expenditure and its components, assessed by a whole-room indirect calorimeter, and *ad libitum* food intake, assessed by using a validated, highly reproducible computerized vending machine paradigm over 3 days. After finding that reduced albumin was associated with greater *ad libitum* food intake, we then evaluated whether albumin concentration predicted future changes in body weight and body composition.

## Methods

### Study Populations

**Figure 1:**
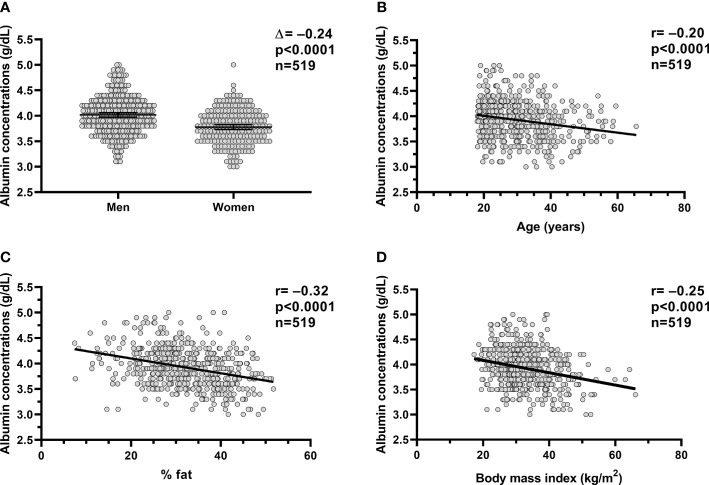
Relationships between plasm albumin concentrations and body composition and age. Relationships between plasma albumin concentration (g/dl) and sex **(A)**, age **(B)**, percent body fat **(C)** and BMI **(D)**. In each panel, the Pearson’s correlation coefficient (r) is reported along with its significance (p). The dots in each panel are referring to the volunteers (n=519) who completed the inpatient part of the studies (**study 1** and **study 2**). %fat, percentage body fat; BMI, body mass index.

The study population included 238 adults, most of whom were Native Americans, admitted to clinical trial NCT00342732 from 1999 to 2017 (*The Food Intake Phenotype: Assessing Eating Behavior and Food Preferences as Risk Factors for Obesity)* (**Study 1**). For this current analysis, we included those (age <65 years) who had valid measures of energy expenditure and substrate oxidation in eucaloric conditions, assessed by whole-room indirect calorimeter (18) and valid measures of *ad libitum* food intake measurement over a 3-day period assessed by a validated, highly reproducible, computerized vending machine paradigm (19) (**Figure 1**, **Supplemental Material**).

The second cohort included 325 adult Native Americans who participated in an ongoing longitudinal clinical trial NCT00340132 (*Cross-sectional and longitudinal studies of “pre-diabetes”)* (**Study 2**) from 1983 to 2009 to evaluate the risk factors for type 2 diabetes and obesity who had follow-up measured of adiposity (**Figure 1**, **Supplemental Material**). The participants were admitted to the clinical research unit as previously described (20). All 325 volunteers completed the inpatient study and returned for follow up visits. A DXA scan for the measurement of body weight and body composition (FM and FFM) was performed at baseline and follow up visit. For the current analysis, we included only those with age ≥ 18 years who were diabetes-free at baseline and follow-up based on oral glucose tolerance test (OGTT).

The volunteers for both study protocols were recruited from the greater Phoenix area and were healthy other than obesity and impaired glucose tolerance, based on medical history and laboratory testing. In particular, the measurement of serum creatinine concentration and electrocardiography were performed to evaluate renal and cardiac function, respectively.

Also, the volunteers were not actively smoking cigarettes or on medications at the time of the inpatient evaluation. Both study protocols were approved by the Institutional Review Board of the National Institute of Diabetes and Digestive and Kidney Diseases (NIDDK). Written and informed consent was signed by the volunteers prior to admission for each study.

In both studies, plasma albumin concentration was measured in the fasting state at admission and was available for all the participants (**Figure 1**, **Supplemental Material**). Volunteers were fed a standard weight-maintaining diet (50% carbohydrate, 30% fat, and 20% protein) upon admission. A unit-specific equation (21) based on body weight and sex was used to calculate the weight maintaining energy needs (WMEN) for each participant and intake was adjusted as needed to ensure a body weight within 1% of the admission weight. After 3 days on a weight-maintaining diet, a 75-g oral glucose tolerance test was performed to assess glucose tolerance according to the American Diabetes Association criteria (22) and subjects with diabetes were excluded.

### Anthropometrics and Body Composition Measurements

In **study 1**, dual-energy x-ray absorptiometry (DPX-1 and DPX-L; Lunar Radiation, Madison, WI) was used to assess body composition (percentage of body fat, %fat) and, hence, to calculate body fat mass (FM) and fat free mass (FFM). Fat free mass and fat mass indexes (FFMI and FMI, respectively) were calculated by dividing the respective masses by height squared. In **study 2**, body composition was assessed by hydro-densitometry (23) or by total body dual-energy X-ray absorptiometry (DPX-L; Lunar Radiation, Madison, WI). The absorptiometry measures were converged to comparable hydro-densitometry values, using previously derived equations (24) to calculate percentage of body fat (%fat). A validated regression equation was used to make DXA data comparable among diverse DXA machine (25) to account for the usage of different DXA machine over the course of the studies. Body mass index (BMI; kg/m2) was calculated by measured height and weight.

### Analytic Measures

The glucose oxidase method (Glucose analyzer GM9, Analox Instruments; Lunenberg, MA; Beckman Instruments Inc., Fullerton, California, USA) was used to measure plasma glucose concentration. Plasma albumin was measured by the bromocresol purple method (Monarch Chemistry analyzer, Instrumentation Laboratory, Lexington, MA; DADE Behring Dimension RxL Chemistry analyzer, Siemens Medical Solutions, Malvern, PA).

### Metabolic Chamber Measures

To measure 24-h energy expenditure (24-h EE), participants of **study 1** spent approximately 23.5 h inside a whole-room indirect calorimeter as previously described (18). To account for reduced physical activity inside the chamber, predefined energy intake in the metabolic chamber was approximately 80% of the weight maintaining diet and based on previously developed equation (26). All unconsumed food was returned to the metabolic kitchen for weighing for an accurate calculation of intake. To calculate 24-h EE and 24-h RQ, carbon dioxide production and oxygen consumption were extrapolated to 24 h. Energy intake minus 24-h EE was defined as 24-h energy balance (ENBAL). The volunteers with ENBAL greater than 20% were excluded from the analysis involving energy expenditure and respiratory quotient. From the 24-h RQ, 24-h carbohydrate oxidation (24-h CARBOX) and lipid oxidation (24-h LIPOX) rates were calculated accounting for 24-h protein oxidation (24-h PROTOX) obtained by the measurement of 24-h urinary nitrogen excretion as previously described (27). Radar sensors were used to detect spontaneous physical activity (SPA) expressed as the percentage of time over the 24-h period in which activity was detected. The average energy expenditure between 2330 and 0500 during which SPA was < 1.5% was defined as sleeping metabolic rate (SMR).

### *Ad Libitum* Food Intake Measures

To assess ad libitum food intake for a 3-day period in participants of **study 1**, a highly reproducible, validated and computerized vending machine paradigm (19, 28) was used. On the admission day, a food selection questionnaire in which a 9-point Likert scale (1=dislike extremely, 5=neutral, 9=like extremely) to assess the food preferences of each participant was given to rate each food item. During the 3-day period, the 40 different food items rated by participants as intermediate with score 4-8 were stocked in an individual-specific, computer-operated vending machines. The volunteers had free access to food for 23.5 h (30 min/day were needed to load the vending machine by the kitchen staff members). Prior to stocking the vending machines, the kitchen staff weighed all food and the leftovers (any uneaten food by the participants) were also weighed at the end of the vending day to precisely estimate the actual intake. The CBORD Professional Diet Analyzer Program (CBORD, Inc., Ithaca, NY, USA) and the Food Processor database (ESHA version 10.0.0, ESHA Research, Salem, OR, USA) were used to calculate the daily total and individual macronutrient kilocalories consumed. The average total *ad libitum* food intake over 3 days was calculated and expressed as total kcal eaten daily. Similar calculations were performed for each macronutrient intake (carbohydrate, fat, and protein). The total energy intake was also expressed as percentage of the WMEN determined prior to the 3-day vending period.

### Statistical Analysis

Statistical analyses were performed using SAS software (SAS 9.3, Enterprise guide version 5.1; SAS Institute, Cary, NC). Data are expressed as mean ± SD or mean with 95% confidence interval (CI). Student’s *t*-test and ANOVA were used to evaluate differences according to gender and ethnicity, respectively. Associations between normally distributed quantitative variables were assessed by the Pearson’s correlation coefficient. To assess the independent determinants of plasma albumin concentrations, we performed multivariate linear regression analysis including age, sex, ethnicity, and body fat as predictors.

Multivariate regression analysis was also performed to assess the associations between plasma albumin concentrations and total *ad libitum* food intake and macronutrient intakes adjusting for their known determinants (i.e., age, sex, ethnicity, FFMI, and FMI) (29). Pearson’s correlation coefficient was then calculated to quantify the relationships between plasma albumin and residual values of food intake measures.

The change in body weight as well as the change in fat mass, fat free mass (expressed in kg) and waist circumference (expressed in cm), were calculated as the difference between the follow-up and the baseline measures recorded during the inpatient admission. Linear regression analysis was first used to calculate adjusted values (i.e., residuals) of changes in body weight, fat mass, fat free mass and waist circumference at follow-up after adjustment for baseline age, sex, and follow-up time. Specifically, adjusted values were calculated after adding the average of each variable to the residuals calculated by the aforementioned regression models.

## Results

Characteristics of the whole study group (**study 1+study 2**) and of each study separately, are reported in **Table 1**. On average, participants were young (30.4 ± 9.3 years) with obesity (33.1 ± 7.9 kg/m^2^). The expected differences in body composition were observed between men and women in both studies, such that women had higher fat mass and lower fat free mass compared to men. In the situation where a few individuals participated in both studies, only the first visit was included for the analysis and reported in the demographic table.

**Table 1:** Demographic and anthropometric characteristics of the combined study cohorts.

	Study 1 + Study 2			Study 1			Study 2		
	(n=519)	Men (n=313)	Women (n=206)	(n=238)	Men (n=92)	Women (n=146)	(n=281)	Men (n=167)	Women(n=114)
**Ethnicity**	11 BLK, 51 WHT, 12 HIS, 421 NAM, 24 O	6 BLK, 38 WHT, 9 HIS, 243 NAM, 17 O	5 BLK, 13 WHT, 3 HIS, 178 NAM, 7 O	11 BLK, 51 WHT, 12 HIS, 140 NAM, 24 O	6 BLK, 38 WHT, 9 HIS, 76 NAM, 17 O	5 BLK, 13 WHT, 3 HIS, 64 NAM, 7 O	281 NAM	167 NAM	114 NAM
**Age (years)**	30.4 ± 9.3	30.9 ± 9.5	29.6 ± 8.8	35.2 ± 10.3	35.5 ± 10.7	34.1 ± 9.6	26.5 ± 6.0	26.8 ± 6.0	26 ± 5.9
**Body weight (kg)**	93.5 ± 23.6	96.2 ± 24	89.3 ± 22.4*	91.5 ± 23.3	93.7 ± 22.7	87.9 ± 23.9	95.2 ± 23.8	98.4 ± 25	90.5 ± 21.1*
**Height (cm)**	167.9 ± 8.6	172.9 ± 6.5	160.4 ± 5.3*	169.5 ± 9.2	174.9 ± 6.6	160.8 ± 5.2*	166.6 ± 7.8	171.1 ± 5.8	160 ± 5.4*
**BMI (kg/m²)**	33.1 ± 7.9	32.1 ± 7.5	34.7 ± 8.3*	31.9 ± 7.9	30.6 ± 6.9	34 ± 8.9*	34.2 ± 7.8	33.5 ± 7.8	35.3 ± 7.7
**Fat free mass (kg)**	62.4 ± 13.2	68.1 ± 11.8	53.6 ± 10.2*	61.9 ± 13.7	67.6 ± 12.1	52.8 ± 11*	62.8 ± 12.8	68.6 ± 11.6	54.3 ± 9.4*
**Fat mass (kg)**	31.1 ± 13.9	28.1 ± 13.6	35.7 ± 13.3*	29.6 ± 13.3	26.1 ± 11.8	35.1 ± 13.7*	32.4 ± 14.4	29.8 ± 14.8	36.2 ± 13*
**Body fat (%)**	32.2 ± 8.7	27.8 ± 7.2	38.9 ± 6.2*	31.3 ± 8.7	26.7 ± 6.7	38.7 ± 6.1*	32.9 ± 8.6	28.8 ± 7.4	39 ± 6.2*
**Fasting glucose (mg/dl)**	90.3 ± 9.2	89.1 ± 9	92.3 ± 9.2*	91.5 ± 7.6	91.3 ± 7.6	91.9 ± 7.6	89.3 ± 10.3	87.1 ± 9.7	92.6 ± 10.4*
**2-h OGTT glucose (mg/dl)**	124.1 ± 28.5	120.7 ± 27.5	129.2 ± 28.6 *	124.3 ± 28.5	120.9 ± 27.4	129.6 ± 29.4	122.2 ± 32.7	117.7 ± 34.7	128.9 ± 28.1
**Albumin (g/dl)^1^**	3.9 ± 0.4	4.0 ± 0.4	3.8 ± 0.3*	3.8 ± 0.3	3.9 ± 0.3	3.6 ± 0.3*	4.0 ± 0.4	4.1 ± 0.4	3.9 ± 0.3*

Data are presented as mean ± SD. *p<0.05 between males and females as determined by unpaired Student’s t-test.

^1^plasma albumin concentration was measured in fasting state (530 AM) on the day of admission.

The values of each variable reported in the table are referring to the volunteers (n=519) who completed the inpatient part of the studies (**study 1** and **study 2**) and when **study 1** and **study 2** were analyzed separately. Of the subjects who completed both studies, the volunteers with the oldest inpatient visit were excluded from the analysis.

BLK, Black; HIS, Hispanic; O, Other; OGTT, oral glucose tolerance test; NAM, Native American; WHT, White.

### Determinants of Plasma Albumin Concentrations

In the whole study group (**study 1**+**study 2)**, plasma albumin concentration measured on the day of admission was on average 3.9 ± 0.4 g/dl and diverged by sex (p<0.0001, **Figure 1A**), such as the plasma albumin concentrations were on average lower in women compared to men by approximately 6% (Δ=−0.24 g/dl, CI: −0.30 to −0.17). Plasma albumin concentrations were negatively associated with age (r=−0.20, p<0.0001, **Figure 1B**), %fat (r= −0.32, p<0.0001, **Figure 1C**), and BMI (r=−0.25, p<0.0001, **Figure 1D**).

In a multivariable model, age (p<0.0001), sex (p=0.0007), and %fat (p<0.0001) were independent determinants of plasma albumin concentrations (total r^2 =^ 0.18) as opposed to ethnicity (p=0.4).

Similar results were observed when **study 1** and **study 2** were analyzed separately.

#### Albumin Concentrations and Energy Expenditure Measures (Study 1)

In the 238 volunteers who completed **study 1** and had a valid assessment of metabolic chamber measures, no associations were observed between plasma albumin concentrations and 24-h EE and 24h RQ, CARBOX, LIPOX, or PROTOX (all p>0.2). Similar results were obtained after adjustment for their known determinants (all p>0.3).

#### Albumin Concentrations and *ad libitum* Food Intake Measures (Study 1)

The *ad libitum* food intake of the participants of **study 1** are reported in **Table 2**. The average, daily, total *ad libitum* energy intake on the vending machines was 3958 ± 1403 kcal/day (or 119 ± 37% when expressed as percentage of WMEN) and was lower in women compared to men by approximately 30%.

**Table 2:** Measures of *ad libitum* food intake by the computerized vending machine system.

	Study 1		
	Whole study group(n=238)	Men (n=146)	Women (n=92)
**Computerized vending machines system**			
**Total energy intake (Kcal/day)^1^**	3958 ± 1403	4354 ± 1352	3329 ± 1251*
**Total energy intake** **(% WMEN)**	119 ± 36	161 ± 48	133 ± 54*
**WMEN (kcal/day)**	2777 ± 254	2856 ± 231	2614 ± 206*
**CHO intake (kcal/day)**	2014 ± 711	2217 ± 686	1692 ± 630*
**FAT intake (kcal/day)**	1514± 636	1660 ± 630	1284 ± 577*
**PRO intake (kcal/day)**	511 ± 192	561 ± 186	433 ± 176*

Data are presented as mean ± SD, unless otherwise indicated.

^1^Ad libitum food intake measures are reported as the average of 3 days on the vending machines. WMEN, weight maintaining energy needs; CHO, carbohydrate. *indicates statistically significant differences between groups.

**Figure 2:**
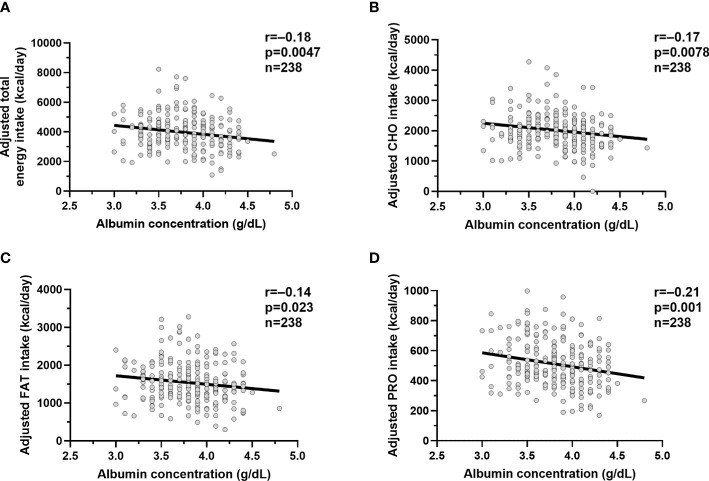
Relationships between albumin concentrations and ad libitum food intake measures. Relationships between plasma albumin concentration (g/dl) and adjusted total *ad libitum* food intake **(A)**, adjusted carbohydrate intake **(B)**, adjusted fat intake **(C)** and adjusted protein intake **(D)**. The total *ad libitum* food intake and each macronutrient intake during the 3-day vending period are expressed as the average over 3 days. The *adjusted* food and macronutrient intake values were calculated *via* linear regression analysis including fat-free mass, fat mass, age, sex, ethnicity after adding the average total food and macronutrient intake to the residual values obtained from the regression model. In each panel, the Pearson’s correlation coefficient (r) is reported along with its significance (p).

Lower plasma albumin concentration was associated with higher total energy intake on the vending machines (β= 89.8 kcal/day per 0.1 g/dl reduction in plasma albumin, p=0.0047, **Figure 2A**), reflecting higher carbohydrate (β= 44.0 kcal/day, p=0.0078, **Figure 2B**), fat (β= 33.8 kcal/day, p=0.023, **Figure 2C**) and protein (β= 13.8 kcal/day, p=0.001, **Figure 2D**) intake after adjustment for their known determinants (age, sex, ethnicity, FMI, and FFMI). Also, lower albumin correlated with higher %WMEN (r=-0.2, p=0.002) after adjustment for age and sex.

We did not observe any difference between plasma albumin and total daily energy intake (adjusted for age, sex, FMI, and FFMI) in the five different ethnic groups (albumin × ethnicity interaction term p=0.12).

#### Albumin Concentrations and Change in Body Composition (Study 2)

**Figure 3:**
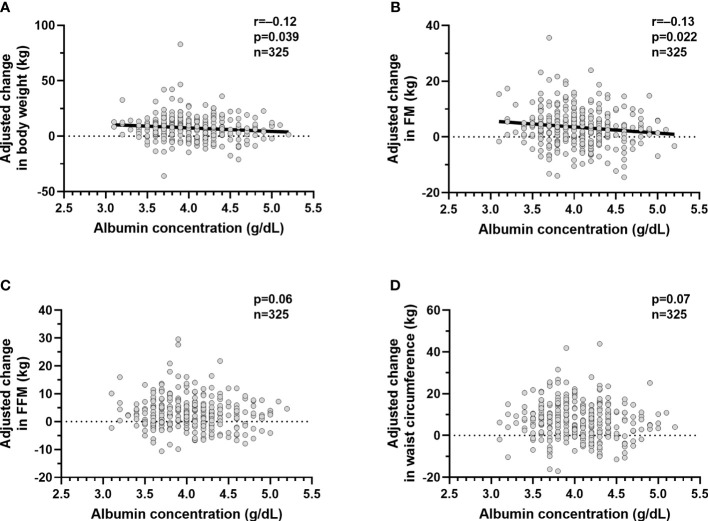
Relationships between plasma albumin concentrations and change in body weight and body composition. Relationships between plasma albumin concentration (g/dl) and adjusted mean change in body weight **(A)**, adjusted mean change in fat mass **(B)**, adjusted mean change in fat free mass **(C)** and adjusted mean change in waist circumference **(D)**. The *adjusted* mean change in body weight, fat mass,fat free mass and waist circumference values were calculated *via* linear regression analysis including age, sex, follow up time in years after adding the average of the mean change in body weight, mean change in fat mass, mean change in fat free mass and mean change in and waist circumference to the residual values obtained from the regression model. In each panel, the Pearson’s correlation coefficient (r) is reported along with its significance (p). The dots represent values for the volunteers who completed the inpatient part of **study 1** (n=325) and who returned for the follow up visit (n=325). The participants recruited were only Native Americans.

Since lower albumin was associated with greater ad libitum food intake, we evaluated whether reduced albumin was associated future weight gain. In **study 2**, mean body weight and BMI at baseline were 64.2 ± 23.4 kg and 33.8 ± 7.7 kg/m^2^, respectively. At the follow up visit after ~6 years (median 5.8 years IQR 2.87 to 7.8), subjects gained on average 7.5 ± 11.7 kg (p<0.0001) reflecting an increase in FM (+3.6 ± 7.4 kg, p<0.0001), FFM (+3.2 ± 5.8 kg, p<0.0001), and waist circumference (+7.2 ± 9.5 cm, p<0.0001). After adjustment for age, sex and follow up time, lower albumin concentrations were associated with increase in weight (β=3.53 kg, p=0.039, CI 0.16 to 6.21 per 1 g/dl difference albumin concentration, **Figure 3A**) and fat mass (β=2.3 kg, p=0.022, CI 0.36 to 4.24, **Figure 3B**), respectively, but not FFM (p=0.06, **Figure 3C**) or waist circumference (p=0.07, **Figure 3D**).

## Discussion

In healthy adults, plasma albumin was not associated with 24-h energy expenditure or substrate oxidation, but lower albumin concentration was associated with greater *ad libitum* energy intake. Lower plasma albumin concentration was also associated with greater weight and fat mass gain over 6 years.

No previous studies have demonstrated that albumin predicts long-term weight change. Since reduced albumin has also been associated with the development of type 2 diabetes (16), the results further suggest that weight gain may be mediating future risk of type 2 diabetes found in prior studies.

Weight gain must be due to reduced energy expenditure or greater energy intake or a combination of the two. We evaluated both sides of the energy balance equation and found that reduced albumin was associated with greater *ad libitum* food intake but not energy expenditure. Though the mechanism linking reduced albumin and weight gain is unclear, these results indicate that reduced albumin may influence appetite regulation. Since lower albumin is associated with higher inflammation (e.g. Il-6) which is known to suppress energy intake (9, 30), we would have expected food intake to be suppressed if the mechanism was related to inflammation. Alternatively, the mechanism may be related to albumin’s ability to binds a host of substances including hormones known to alter appetite (e.g. thyroxine, glucocorticoids) (31–33). Albumin may serves as a carrier-ligand for these substances providing transport to sites of action, metabolism, and excretion (34). Albumin-ghrelin binding has been shown to impair ghrelin biological activity (17). Reduced circulating albumin may result in less albumin-ghrelin interaction and thus greater ghrelin availability and activity of ghrelin stimulating appetite (17). Albumin also binds fatty acids (35) which have been implicated as signaling molecules for hypothalamic appetite regulation (36). Oleic acid (OA), a monounsaturated peripheral circulating fatty acid, has been shown to affect food intake in rodent models. Central administration of OA reduced food intake (37–39) through the increase in the gene expression of pro-opiomelanocortin (POMC) which is a known anorexigenic pathways. Although there is high affinity of albumin for fatty acids in circulation, experimental findings indicate that albumin may facilitate availability of these substrates at target organs (40). We might then also speculate that decreased circulating albumin might influence energy intake *via* fatty acids as a signal for nutrient availability. Thus, potential mechanisms may be related to albumin’s carrier functions or interactions with hormones known to regulate appetite such as ghrelin and other molecules such as fatty acids.

We also evaluated the anthropometric determinants of plasma albumin concentration. Few studies in literature have investigated the relationship between albumin and body composition.

In elderly patients hospitalized in a rehabilitation unit, serum albumin was not associated with lean mass or appendicular skeletal muscles mass (41). Recently, two recent studies showed a negative correlation between serum albumin and BMI (42, 43). Also, it has been shown that plasma albumin concentration is negatively associated with percentage body fat in Native American population (16). In line with these latter findings, we confirmed that lower plasma albumin is associated with higher percent of body fat, but not with fat free mass.

One of the strengths of the present study is the large cohort of healthy participants, allowing this analysis to fully examine circulating plasma albumin concentrations as a predictor of weight change and as a predictor of short-term *ad libitum* intake in humans. Furthermore, the measurements of energy metabolism and *ad libitum* food intake were performed in a carefully controlled inpatient setting using very accurate and highly reproducible methods including measurements of EE over 24 h in a whole-room indirect calorimeter, as well as measurements of *ad libitum* food intake over 3 days using computerized vending machines.

Despite the strengths, our study has several limitations. Only baseline albumin was measured, thus the association between albumin and body weight change might be altered after accounting for repeated measurement over time. Furthermore, the study did not evaluate orexigenic/anorexigenic hormones or other serum inflammatory markers besides albumin. Moreover, although the correlation coefficients were not strong, the statistically significant associations indicate that the study had sufficient sample size to detect these associations.

Finally, we could not assess *in vivo* interactions between albumin and hormones known to influence appetite, so the precise mechanism remains unclear.

## Conclusion

In conclusion, in a large cohort of overweight but otherwise healthy subjects, lower albumin concentration was associated with future weight and fat mass gain. This may be due to an increase in *ad libitum* food and macronutrient intake, also associated with plasma albumin levels. These results suggest that albumin might be considered a marker of energy intake playing a role in the regulation of energy balance equation and in regulating weight change and possibly suggesting a biological reason for the “non adherence” to dietary prescriptions in persons with obesity.

## Data Availability Statement

The raw data supporting the conclusions of this article will be made available by the authors, without undue reservation.

## Ethics Statement

The studies involving human participants were reviewed and approved by National Institute of Diabetes and Digestive and Kidney Diseases, National Institutes of Health (NIH). The patients/participants provided their written informed consent to participate in this study.

## Author Contributions

AB and TA analyzed and interpreted data and wrote the manuscript. DC, PP, and TH assisted with the interpretation of the data and revised the manuscript. SV and JK designed the two clinical protocols, supported with the interpretation of the data, and reviewed the manuscript. SV edited the manuscript and interpreted data. All authors contributed to the article and approved the submitted version. AB has full access to all the data in the study and take responsibility for the integrity of the data and the accuracy of the data analysis.

## Funding

This work was supported by the Intramural Research Program of the National Institutes of Health, National Institute of Diabetes and Digestive and Kidney Diseases. This research was supported by the Intramural Research Program of the NIH, The National Institute of Diabetes and Digestive and Kidney Diseases (NIDDK). PP was supported by the program “Rita Levi Montalcini for young researchers” from the Italian Minister of Education and Research.

## Conflict of Interest

The authors declare that the research was conducted in the absence of any commercial or financial relationships that could be construed as a potential conflict of interest.

## Abbreviations

DXA, dual energy X-ray absorptiometry; FFM, fat free mass; FFMI, fat free mass index; FM, fat mass; FMI, fat mass index; OGTT, oral glucose tolerance test; %FAT, percent of body fat; WC, waist circumference; 24-h EE, 24-h energy expenditure; ENBAL, 24-h energy balance; SMR, sleeping metabolic rate; 24h RQ, 24-h respiratory quotient; 24h CARBOX, 24-h carbohydrate oxidation; LIPOX, 24-h lipid oxidation; PROTOX, 24-h protein oxidation; SPA, spontaneous physical activity; WMEN, weight maintaining energy needs.
