# Supplementary material for: Reduced Albumin Concentration Predicts Weight Gain and Higher Ad Libitum Energy Intake in Humans
Source: Front Endocrinol (Lausanne). 2021 Mar 11;12:642568. doi: 10.3389/fendo.2021.642568 (PMC7991842; doi:10.3389/fendo.2021.642568)
Supplement: Supplementary file 1 [file DataSheet_1.pdf]

Supplementary Material

Flow chart of the study population

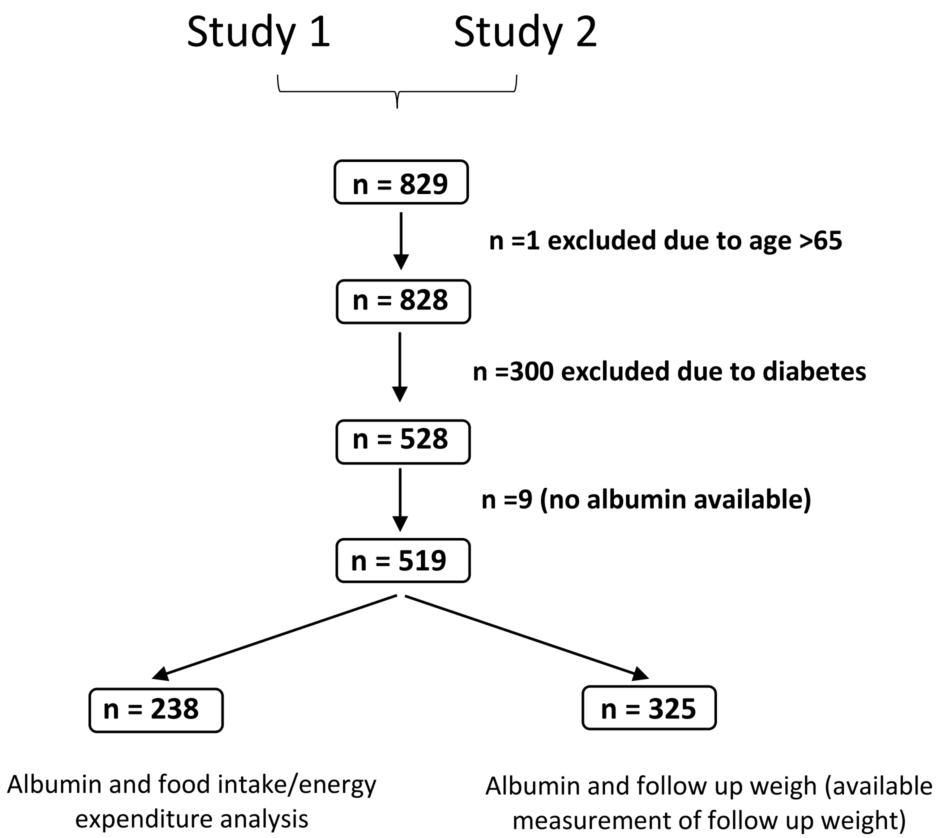

The flow chart shows the study population used for the analysis.
Since some volunteers completed both studies, we chose to keep only the data of that subject who completed the study 2 which was the study with follow up weight available (study 2). Thus, the analysis between plasma albumin concentration and body composition was performed in 519 participants. Of those 519 volunteers, 238 of the study 1 had available measurement of food intake and energy expenditure (as shown in Fig.1). On the other hand, the analysis between albumin and follow up weight was performed only in the study 2 (n=325) which was the study with available follow up weight.
